# MmpA, a Conserved Membrane Protein Required for Efficient Surface Transport of Trehalose Lipids in Corynebacterineae

**DOI:** 10.3390/biom11121760

**Published:** 2021-11-24

**Authors:** Tamaryn J. Cashmore, Stephan Klatt, Rajini Brammananth, Arek K. Rainczuk, Paul K. Crellin, Malcolm J. McConville, Ross L. Coppel

**Affiliations:** 1 Infection and Immunity Program, Monash Biomedicine Discovery Institute and Department of Microbiology, Monash University, Melbourne, VIC 3800, Australia; tamcashmore@gmail.com (T.J.C.); rajini.brammananth@monash.edu (R.B.); alecrain@gmail.com (A.K.R.); ross.coppel@monash.edu (R.L.C.); 2 Department of Biochemistry and Pharmacology, Bio21 Institute of Molecular Sciences and Biotechnology, University of Melbourne, Parkville, VIC 3010, Australia; klatt@vrc.uni-frankfurt.de (S.K.); malcolmm@unimelb.edu.au (M.J.M.)

**Keywords:** *Mycobacterium* *tuberculosis*, *Corynebacterium* *glutamicum*, cell wall, lipid bilayer, glycolipid, trehalose monohydroxycorynomycolate (hTMCM), trehalose monoacetylcorynomycolate (AcTMCM)

## Abstract

Cell walls of bacteria of the genera *Mycobacterium* and *Corynebacterium* contain high levels of (coryno)mycolic acids. These very long chain fatty acids are synthesized on the cytoplasmic leaflet of the inner membrane (IM) prior to conjugation to the disaccharide, trehalose, and transport to the periplasm. Recent studies on *Corynebacterium glutamicum* have shown that acetylation of trehalose monohydroxycorynomycolate (hTMCM) promotes its transport across the inner membrane. Acetylation is mediated by the membrane acetyltransferase, TmaT, and is dependent on the presence of a putative methyltransferase, MtrP. Here, we identify a third protein that is required for the acetylation and membrane transport of hTMCM. Deletion of the *C. glutamicum* gene *NCgl2761* (*Rv0226c* in *Mycobacterium tuberculosis*) abolished synthesis of acetylated hTMCM (AcTMCM), resulting in an accumulation of hTMCM in the inner membrane and reduced synthesis of trehalose dihydroxycorynomycolate (h2TDCM), a major outer membrane glycolipid. Complementation with the *NCgl2761* gene, designated here as *mmpA*, restored the hTMCM:h2TDCM ratio. Comprehensive lipidomic analysis of the Δ*tmaT*, Δ*mtrP* and Δ*mmpA* mutants revealed strikingly similar global changes in overall membrane lipid composition. Our findings suggest that the acetylation and membrane transport of hTMCM is regulated by multiple proteins: MmpA, MtrP and TmaT, and that defects in this process lead to global, potentially compensatory changes in the composition of inner and outer membranes.

## 1. Introduction

Bacteria of the genera *Mycobacterium* and *Corynebacterium* comprise the suborder Corynebacterineae and cause severe human diseases such as tuberculosis (*Mycobacterium tuberculosis*), leprosy (*Mycobacterium leprae*) and diphtheria (*Corynebacterium diphtheriae*). *M. tuberculosis* latently infects around one-quarter of the human population, resulting in ~1.4 million deaths annually from tuberculosis (TB) [1]. Around ten million people contracted TB in 2019, mostly in Southeast Asia, Africa and the Western Pacific region. While the incidence of TB has been gradually falling, the prevalence of drug resistant strains of *M. tuberculosis*, particularly rifampicin-resistant (RR-TB) and multi-drug resistant (MDR-TB) strains, is increasing and represents a significant threat. Several first line TB drugs, such as isoniazid and ethambutol, target synthesis of the unique, lipid-rich cell wall of these bacteria and an improved understanding of the processes by which the cell wall is synthesised and assembled is expected to reveal new targets for the development of new antimicrobials.

The multi-laminate cell wall of corynebacteria and mycobacteria increases the resistance of these bacteria to many antibiotics and protects against host microbicidal responses. The inner layers of the cell wall comprise peptidoglycan and the polysaccharide, arabinogalactan (AG). AG is covalently modified with very long chain (70–90 carbon) fatty acids, the (coryno)mycolic acids, that form the inner leaflet of an outer membrane (OM) [2,3,4]. These α-alkyl β-hydroxyl fatty acids are either linked to terminal arabinose residues of the AG polymer or are present as free lipids esterified to trehalose (trehalose monomycolate (TMM) and trehalose dimycolate (TDM)), glucose (glucose monomycolate (GMM)) or glycerol (glycerol monomycolate (GroMM)) [5].

The key steps in the biosynthesis of the mycolic acids (in mycobacteria) and structurally related (coryno)mycolic acids (in corynebacteria) have been intensively studied (see reviews [4,6,7]). The first committed intermediate in mycolic acid biosynthesis, a 2-alkyl-3-keto fatty acid, is generated via the Claisen condensation of two fatty acids catalysed by the cytoplasmic polyketide synthase, Pks13 [8]. The short alpha branch (C_24_-C_26_) of this intermediate is the product of enzyme FAS-I [9], while the long meromycolate chain (C_50_-C_60_) is the product of the FAS-II complex [10]. The 2-alkyl 3-keto fatty acid is subsequently reduced by CmrA to generate the corresponding hydroxylated mycolic acid [11] that is conjugated to trehalose to form the abundant cell wall component TMM. TMM is transported across the inner membrane (IM) by the general lipid transporter MmpL3 [12,13] where it serves as a general mycolic acid donor. In particular, periplasmic mycolyltransferases of the Ag85ABC complex [14] transfer mycolic acids from TMM to acceptors in the AG layer, to other TMM molecules to form TDM, or to glucose or glycerol.

While the enzymatic reactions of mycolic acid biosynthesis are well understood, less is known about the transport of TMM intermediates across the IM. We have addressed this question in recent studies using *Corynebacterium glutamicum*. This organism serves as a useful model because it can tolerate cell wall defects that render Mycobacteria non-viable, allowing mutants to be produced for characterisation [11,15,16,17]. We recently showed that the hydroxylated corynomycolate chain in *C. glutamicum*, trehalose monocorynomycolate (hTMCM), is transiently modified with an acetyl group and that this modification is important for periplasmic transport of TMCM. Trehalose monoacetylcorynomycolate (AcTMCM), is synthesized by the acetyltransferase TmaT (NCgl2759). Genetic deletion of *tmaT* significantly reduced the rate of corynomycolate transport across the IM and synthesis to trehalose dihydroxycorynomycolate (h2TDCM) [17], indicating that AcTMCM is the major substrate transported by *C. glutamicum* transporters. A second protein, annotated as a putative methyltransferase, MtrP (NCgl2764), was subsequently shown to be required for AcTMCM synthesis and membrane transport in addition to TmaT [18]. Here, we provide evidence that a third protein, encoded by the same gene locus, is also required for this process. Disruption of a gene encoding a putative integral membrane protein, *NCgl2761,* which we designate as *mmpA*, also lead to the accumulation of hTMCM on the cytoplasmic face of the IM and reduced synthesis of h2TDCM. A comprehensive lipidomic analysis of wild-type (WT) and mutant strains showed that MmpA is required for synthesis of AcTMCM and that loss of *tmaT*, *mtrP* and *mmpA* leads to similar global changes in the levels of other inner and outer membrane lipids. These findings indicate that multiple proteins regulate the acetylation and balanced transport of trehalose corynomycolates across the IM of these bacteria.

## 2. Materials and Methods

### 2.1. Bacterial Strains and Culturing Conditions

*Escherichia coli* DH5α was grown in Luria-Bertani (LB) medium at 37 °C with aeration. *C. glutamicum* ATCC 13032 was grown in Brain Heart Infusion (BHI) medium (Oxoid) or LBHIS (LB, BHI, sorbitol) at 30 °C with aeration. When necessary, ampicillin was added to a final concentration of 100 µg mL^−1^ and kanamycin at 50 µg mL^−1^. 

### 2.2. Genetic Manipulation of Bacteria

*E. coli* plasmid DNA was isolated using the High Pure plasmid isolation kit (Roche) and *C. glutamicum* genomic DNA was extracted using the Illustra DNA extraction kit (GE Healthcare), according to the manufacturer’s instructions. DNA manipulations and molecular biology techniques were performed as described [17]. 

### 2.3. Bioinformatics

The corynebacterial ortholog of mycobacterial Rv0226c was found using the BLASTp [19] algorithm. Mycobacterial DNA and protein sequences were obtained from Mycobrowser (https://mycobrowser.epfl.ch, accessed on 1 July 2019). Protein topology predictions were performed using Protter v1.0 [20] (http://wlab.ethz.ch/protter/start/, accessed on 8 September 2020). Amino acid sequence alignments were generated using Clustal Omega (http://www.ebi.ac.uk/Tools/msa/clustalo/, accessed on 1 July 2019). Structural model of NCgl2761 was generated using Alphafold2 (https://alphafold.ebi.ac.uk, accessed on 7 October 2021) [21].

### 2.4. Reverse Transcriptase (RT)-PCR

To prepare RNA, bacterial pellets were suspended in 20 mM NaOAc, 0.05% (*w*/*v*) SDS, 1 mM EDTA and 100 μg proteinase K (pH 4.0) then agitated with acid-washed glass beads in a bead beater. After centrifugation, the supernatant was extracted with phenol:chloroform:isoamyl alcohol (25:24:1 *v*/*v*) then re-extracted with chloroform:isoamyl alcohol (24:1 *v*/*v*). The upper phase was precipitated with an equal volume of ice-cold isopropanol followed by incubation at −70 °C for 30 min. After centrifugation (30 min, 4 °C) the pellet was washed with 70% ethanol, dried under vacuum, and resuspended in water. DNaseI treatment was performed by adding RNasin, 10X RQ1 buffer and RQ1 DNase1 (Promega) at 37 °C for 20 min. The RNA was then purified using RNeasy Purification columns (Qiagen). To synthesize cDNA from RNA, the Transcriptor High Fidelity cDNA Synthesis Kit (Roche) was used according to the manufacturer’s instructions. cDNA was then used as the template in a PCR reaction using primers RT1 (5′-CGTATTGGGTGAGGATTCC) and RT2 (5′-AACTCGCCACGCCCGTGAC) with the following program: 94 °C, 8 min; 35 cycles of 94 °C 15 s, 63 °C 1 min, 72 °C 3 min; then 72 °C 7 min. 

### 2.5. Construction of C. glutamicum ΔNCgl2761 and Complementation Strains

A 1.0 kb fragment containing sequence from the 5′ “left” side of the *NCgl2761* gene was amplified using ProofStart DNA polymerase (Qiagen) with primers *NCgl2761*-left_F (5′- ATCCCCGGGTTTGGAATGCAACGTGG) and *NCgl2761*-left_R (5′- ATCGGATCCCACAGTCGTCCATGCC) and cloned into the XmaI/BamHI sites (underlined) of pUC19 [22], creating plasmid pUC-*NCgl2761*left. A 1.0 kb fragment containing sequence from the 3′ “right” side of the *NCgl2761* gene was amplified using primers *NCgl2761*-right_F (5′- ATGGGATCCCGGTTGTCG) and *NCgl2761*-right_R (5′- TACTCTAGA-CGTGCCGCTGCACAC) and cloned into the BamHI/XbaI sites (underlined) of pUC19, creating plasmid pUC-*NCgl2761*right. The right flanking sequence was then liberated from pUC-*NCgl2761*right using BamHI/XbaI and subcloned into Xba/BamHI-digested pUC-*NCgl2761*left, fusing the left and right flanking sequences to create plasmid pUC-Δ*NCgl2761*. The 2.0 kb insert was then liberated using XmaI/XbaI and subcloned into XmaI/XbaI-digested pK18*mobsacB*, a suicide plasmid for *C. glutamicum* [23] that contains kanamycin selection and sucrose contra-selection markers. The resultant plasmid, pK18*mobsacB*:Δ*NCgl2761*, was sequenced then electroporated into electrocompetent *C. glutamicum* cells, prepared as previously described [24], using an ECM 630 electroporator (BTX). Clones resulting from single homologous recombination events were selected on kanamycin. These were grown overnight without antibiotic selection then serially diluted and plated onto LBHIS plates containing 10% (*w*/*v*) sucrose to select for a second crossover event. Sucrose-resistant, kanamycin-sensitive colonies were screened by PCR using NCgl2761-left_F and NCgl2761-right_R primers, with potential *NCgl2761* mutants confirmed using Southern blot hybridization.

To complement the Δ*NCgl2761* strain, the *NCgl2761* gene plus 203 bp of upstream sequence was PCR amplified using primers NCgl2761-comp_F (5′- TCGCCCGGGTA-TACTGCGGATACGTTGAAGCTTCTGC) and NCgl2761-comp_R (5′- TCGCCCGGG-AACTACAAGTGGGAC), digested with XmaI (underlined) and cloned into the unique PvuII site of pSM22 [25], which contains the corynebacterial origin of replication *repA* and kanamycin resistance gene *aphA3.* A sequenced complementation plasmid (pSM22:*NCgl2761*) and pSM22 control plasmid were electroporated into the *C. glutamicum* Δ*NCgl2761* deletion strain, followed by selection on kanamycin supplemented BHI plates.

### 2.6. Southern Hybridization

For Southern blot analysis, 2 µg of genomic DNA was digested with appropriate restriction enzymes under optimal conditions for 16 h. Purified samples and digoxygenin (DIG)-labelled, HindIII-digested λ DNA markers, were separated on a 1% agarose gel, followed by depurination, denaturation, neutralisation, and capillary transfer onto a nylon membrane. The membrane was then hybridised at 65 °C with a gene-specific probe prepared by DIG labelling a 1.6 kb PCR product obtained using primers *NCgl2761*-comp_F and *NCgl2761*-comp_R.

### 2.7. Extraction of Cell Wall Components, HPTLC and SDS-PAGE

*C. glutamicum* strains were grown to exponential phase (OD_600nm_ = 5–7) in BHI medium (Oxoid). Total lipids were extracted in chloroform:methanol (2:1 *v*/*v*) and chloroform:methanol:water (1:2:0.8, *v*/*v*). After removal of insoluble material by centrifugation (15,000× *g*, 10 min), extracts were dried under nitrogen and subjected to biphasic partitioning in 1-butanol: water (2:1 *v*/*v*). The organic phase was dried, and lipids were resuspended in 1-butanol. Cell surface exposed lipid was extracted by water-saturated 1-butanol at RT for 30 min followed by the residual lipid extraction by chloroform:methanol as described for total lipid extraction. The lipid fraction was analysed by high- performance thin-layer chromatography (HPTLC) using aluminium-backed silica gel sheets (Merck). One-dimensional HPTLCs were developed in chloroform: methanol:13 M ammonium solution:1 M ammonium acetate: water (180:140:9:9:2.3 *v*/*v*). Glycolipids were stained and visualized with orcinol/H_2_SO_4_.

### 2.8. Lipoglycan Extraction and Analysis

LM and LAM were extracted and purified as previously described [26], separated by polyacrylamide gel electrophoresis (PAGE) and stained using a SilverSnap kit (Pierce). Alternatively, purified LM/LAM was suspended in 10 mM ammonium carbonate/bicarbonate buffer (pH 8.16) and 4 µL was injected by direct infusion into an Agilent 6220 Accurate-Mass Time-of-Flight LC/MS system (Agilent Technologies, Santa Clara, CA, USA). The run was performed in negative ionization mode, with a mass range of 100–3000 Da. The reference nebulizer was set to 20 psig with a detection window of 100 ppm, a minimum height of 1000 counts, an acquisition rate of 0.63 spectra/s, and an acquisition time of 1589.4 ms/spectrum. The gas temperature was set to 325 °C with a drying gas of 7 L/min, dual ESI 3500 V, Fragmentor 150 V, and Skimmer 65 V. The flow rate was set to 0.25 mL/min, and a solvent consisting of 0.1 M formic acid, acetonitrile (1:1, *v*/*v*) was used to wash the lines for 25 min. Data were analysed using MassHunter and the length of the mannan chain was calculated, assuming the presence of a PI lipid anchor containing C16:1/18:1/16:0 fatty acids. Analyses were performed in triplicate.

### 2.9. Extraction of IM and OM Lipids for LC/MS and Lipidomics Analyses

IM and OM lipids of *C. glutamicum* were extracted and analysed as previously described [27]. In brief, four replicates of WT *C. glutamicum* and mutant strain were grown to the exponential phase (OD_600nm_ = 2.5–3) in BHI medium and the cells were harvested by centrifugation. The cell pellet was extracted with water-saturated 1-butanol to selectively remove OM lipids. After another centrifugation step, the same pellet was sequentially extracted in chloroform:methanol (2:1, *v*/*v*) and chloroform:methanol:water (1:2:0.8 *v*/*v*) to extract the remaining lipids of the IM. Both IM and OM lipids were separated on an Agilent 1290 Infinity Quaternary LC System (Agilent Technologies) using a C18 column (Phenomenex Kinetex, 2.6 um EVO C18 100A) eluted with an isopropanol mobile phase binary solvent system at a flow rate of 260 µL/min. Eluted lipids were analysed on a 6550 iFunnel Q-TOF LC/MS instrument (Agilent Technologies) in positive ionization mode. Lipids were identified based on their mass-to-charge ratio and fragmentation pattern using a lipid library [27]. Statistical analyses were performed using MetaboAnalyst. 

### 2.10. Metabolic Labeling 

Mid-log phase cultures of *C. glutamicum* were pelleted, metabolically labelled with ^14^C-acetate and sequentially extracted in 1-butanol (BuOH) and chloroform:methanol (C/M; 2:1 *v*/*v*) as previously described [17]. Bands were quantified using ImageJ software (https://imagej.nih.gov/ij, accessed on 2 February 2021).

## 3. Results

### 3.1. Identification of the Corynebacterial Ortholog of M. Tuberculosis Rv0226c

**Figure 1:**
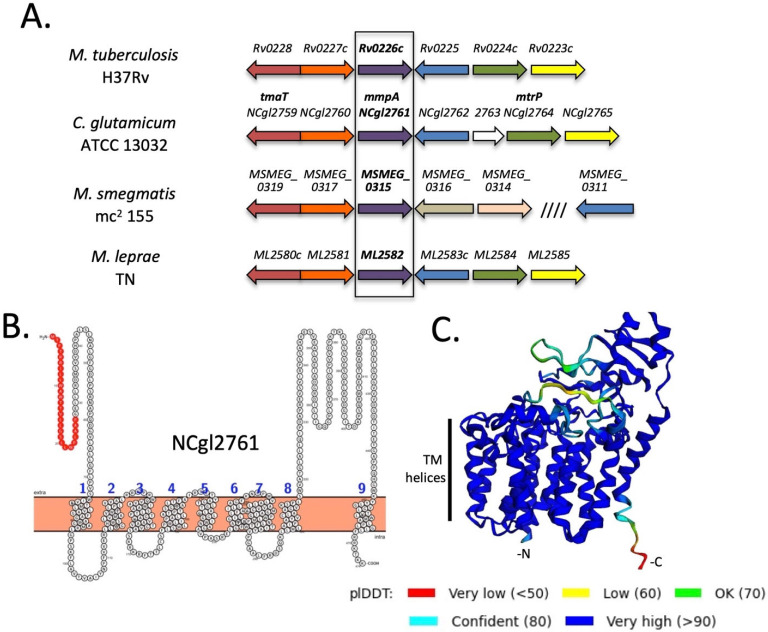
NCgl2761 is a predicted integral membrane protein encoded within a conserved genetic locus highly conserved in Corynebacterineae. (**A**) The *tmaT* locus of Corynebacterineae. Likely orthologous genes in the four species are shown using the same colour. The focus of the current study *NCgl2761* and its mycobacterial orthologs are in bold. Previously studied genes are *NCgl2759* (*tmaT*; [17]), *NCgl2764* (*mtrP*; [18]) and *NCgl2760* [26] while the remaining genes are uncharacterized. (**B**) Protter prediction [20] of the membrane topology of NCgl2761. Numbers indicate predicted transmembrane helices while the putative N-terminal signal sequence is red. (**C**) Structural model of NCgl2761 generated using the Alphafold2 AI system [21] reveals a multi-pass membrane protein with a single non-membrane domain near the C-terminus. Colors represent predicted IDDT confidence values. The N-terminus (-N), C-terminus (-C) and putative transmembrane helices are labelled.

We have previously identified a gene locus that is highly conserved in mycobacteria (including *M. tuberculosis*, *M. leprae* and *M. smegmatis*) and corynebacteria and is important for cell wall synthesis (Figure 1A). In particular, biochemical analysis of *C. glutamicum* mutant lines lacking individual genes in this locus (*NCgl2759*-*2765*) indicate that NCgl2759 and NCgl2764 are involved in the maturation of hTMCM and transport of this glycolipid across the inner bacterial membrane. *NCgl2759* encodes the acetyltransferase, TmaT, that acetylates the hydroxyl group on the corynomycolic acid chain of hTMCM facilitating the transport of this glycolipid by the membrane transporters CmpL1 and CmpL4 [17]. NCgl2764 has sequence homology to methyltransferases and is also required for AcTMCM synthesis and corynomycolate transport, although its precise function is unknown [18]. In contrast, loss of *NCgl2760* (*Rv0227c* in *M. tuberculosis*) had no effect on TMCM/TDCM biosynthesis, but led to a defect in the synthesis of major cell wall components lipomannan (LM) and lipoarabinomannan (LAM) [26]. The next gene of the locus, *NCgl2761*, is also conserved in all mycobacterial/corynebacterial species and its homolog in *M. tuberculosis* H37Rv, *Rv0226c*, is reported to be essential for bacterial growth [28,29,30] and encode a protein that localizes to the cell membrane fraction [31]. However, it remains unclear whether *NCgl2761*/*Rv0226c* is involved in TMCM/TDCM or LM/LAM biosynthesis, or other pathways of cell wall synthesis. BLASTp analysis showed that *NCgl2761* is predicted to encode a protein of 474 residues, sharing 35% identity and 46% similarity with *M. tuberculosis* Rv0226c (Figure S1). It is predicted to be a polytopic membrane protein consisting of a signal peptide and nine transmembrane domains with a significant extracytoplasmic domain between transmembrane helices 8 and 9 (Figure 1B). However, the putative signal sequence may in fact comprise a tenth transmembrane domain, as predicted by the Alphafold2 artificial intelligence system (Figure 1C) [21]. While well conserved amongst the Corynebacterineae, NCgl2761/Rv0226c shares no significant sequence similarity with any functionally characterised protein from other bacteria.

### 3.2. Evidence of NCgl2760 and NCgl2761 Co-Transcription

**Figure 2:**
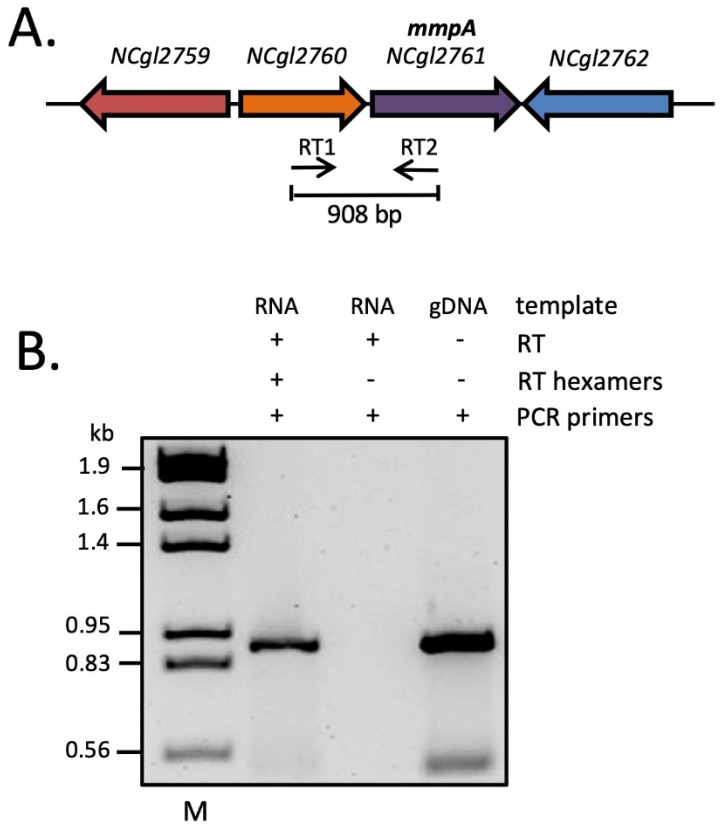
RT-PCR analysis reveals co-transcription of *NCgl2760* and *NCgl2761*. (**A**) The genetic locus with binding sites and orientations of the two RT-PCR primers within the two genes indicated by thin horizontal arrows. (**B**) Agarose gel electrophoresis of RT-PCR reaction products. RNA was prepared from WT *C. glutamicum* and incubated with reverse transcriptase (RT) in the presence of random hexamers or in their absence (negative control). The samples were then subjected to a PCR using primers RT1 and RT2 to detect complementary DNA (cDNA) that spans both genes. Purified *C. glutamicum* genomic DNA (gDNA), PCR amplified using the same primer pair, was used as a positive control. M, λHindIII/EcoRI DNA size markers (indicated in kb).

The tandem genetic arrangement and presence of just 6 bp separating the stop and start codons of the LAM biosynthesis gene *NCgl2760* [26] and *NCgl2761* raised the possibility of co-regulation at the transcriptional level. To investigate co-transcription of the two genes, a reverse transcriptase polymerase chain reaction (RT-PCR) was performed on total RNA isolated from WT *C. glutamicum* using primers designed to bind in the middle of each gene (Figure 2A). A product of the expected size was detected when all reaction components were included (Figure 2B, lane 2). An identical reaction in which the random hexamers essential for conversion of RNA to cDNA were omitted gave no product (lane 3), ruling out the possibility of genomic DNA contamination of the RNA sample. As expected, a positive control in which *C. glutamicum* genomic DNA was amplified using the same primer pair gave a product of identical size (lane 4). These findings provide evidence of co-transcription of *NCgl2760* and *NCgl2761*, raising the possibility that NCgl2761 also functions in LM/LAM biosynthesis since linked genes often have related functions. 

### 3.3. Inactivation of the NCgl2761 Gene Affects Growth of C. glutamicum

**Figure 3:**
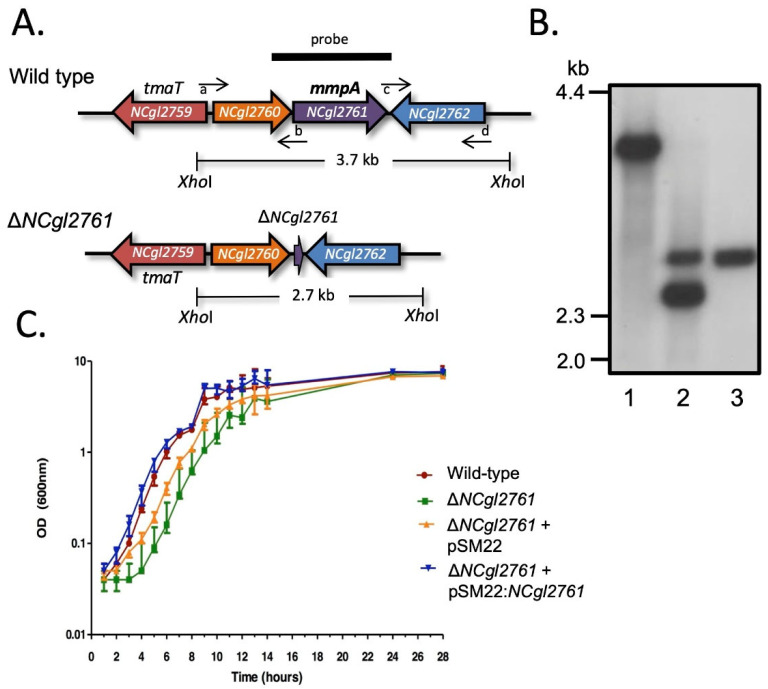
Disruption strategy and growth assessment of *C. glutamicum* Δ*NCgl2761*. (**A**) Diagram showing the arrangement of genes in the *NCgl2761* region of WT *C. glutamicum* (above) and Δ*NCgl2761* mutant (below). XhoI restriction sites, sizes of expected bands on Southern blot and position of the probe used are shown. Small horizontal arrows indicate the binding sites for the four primers used to construct the Δ*NCgl2761* mutant (a, *NCgl2761*-left_F; b, *NCgl2761*-left_R; c, *NCgl2761*-right_F; d, *NCgl2761*-right_R). (**B**) Southern blot analysis of XhoI-digested DNA of *C. glutamicum* WT (lane 1), a single cross-over strain (lane 2) and the Δ*NCgl2761* mutant (lane 3). Positions of DIG-labelled λDNA standards digested with HindIII are indicated in kilobase pairs (kb). (**C**) Growth curves of WT *C. glutamicum*, the Δ*NCgl2761* mutant and complementation strains in liquid BHI medium. Each strain was grown to saturation, then diluted 1:100 in BHI medium. Triplicate cultures were sampled at the times indicated to determine the optical density (OD) at a wavelength of 600 nm. Growth curves were plotted as mean ± SD.

To investigate whether *NCgl2761* is required for either TMCM/TDCM and/or LM/LAM biosynthesis, a *C. glutamicum* null mutant was generated using a two-step allelic replacement strategy previously used in our laboratory to create cell wall mutants [11,15,16,17,27] (see Methods; Figure 3A). Sequences flanking the gene were PCR amplified and cloned into the suicide vector pK18*mobsacB* [25], which carries a kanamycin resistance gene (*aph*) and *Bacillus subtilis sacB* gene conferring sensitivity to sucrose. This plasmid was introduced into *C. glutamicum* ATCC 13032 and potential *NCgl2761* deletion mutants were identified by PCR screening (data not shown), then confirmed by Southern blot analysis. A probe derived from *NCgl2761* hybridized to a 3.7 kb XhoI fragment in genomic DNA from the *C. glutamicum* parent and to a 2.7 kb fragment in a potential Δ*NCgl2761* mutant (Figure 3B). These were the expected profiles (Figure 3A), with the smaller hybridizing fragment in the mutant resulting from deletion of 1.0 kb internal to the gene (Figure 3A,B). As observed for Δ*NCgl2759* [17] and Δ*NCgl2764* [18] mutants, but not for a Δ*NCgl2760* mutant [26], the Δ*NCgl2761* strain had an extended lag-phase of growth compared to the WT parent strain in liquid BHI medium (Figure 3C). To complement the mutant, Δ*NCgl2761* was transformed with pSM22:*NCgl2761*, a shuttle vector carrying a full-length *NCgl2761* gene plus 203 bp of upstream sequence which may include the native promoter. The empty pSM22 vector was also introduced into Δ*NCgl2761* as a control. Growth curves indicated that the introduction of pSM22:*NCgl2761* into the Δ*NCgl2761* mutant removed the extended lag phase, while the empty pSM22 vector did not restore growth to the same extent (Figure 3C). 

### 3.4. Loss of NCgl2761 Does Not Impact Lipoglycan Biosynthesis

Since our RT-PCR data demonstrated co-transcription of the LM/LAM biosynthesis gene, *NCgl2760*, and *NCgl2761*, we first examined the LM/LAM composition of the Δ*NCgl2761* mutant. Delipidated pellets of the mutant and control strains were treated with KOH to extract polar lipoglycans for analysis by SDS-PAGE and silver staining. As expected, the previously characterized Δ*NCgl2760* mutant line synthesized truncated (t-LM, [26]) lipoglycans that exhibited a faster mobility in SDS-PAGE than the LM/LAM species produced by WT bacteria. In contrast, the Δ*NCgl2761* mutant synthesized LM/LAM species that were indistinguishable from WT, when analysed by SDS-PAGE and ESI-TOF-MS profiling (Figure S2). These results suggest that *NCgl2761* is not directly required for LM/LAM biosynthesis despite the co-transcription of *NCgl2760* and *NCgl2761*.

### 3.5. Loss of NCgl2761 Changes the Glycolipid Profile and Distribution of Trehalose Corynomycolates

**Figure 4:**
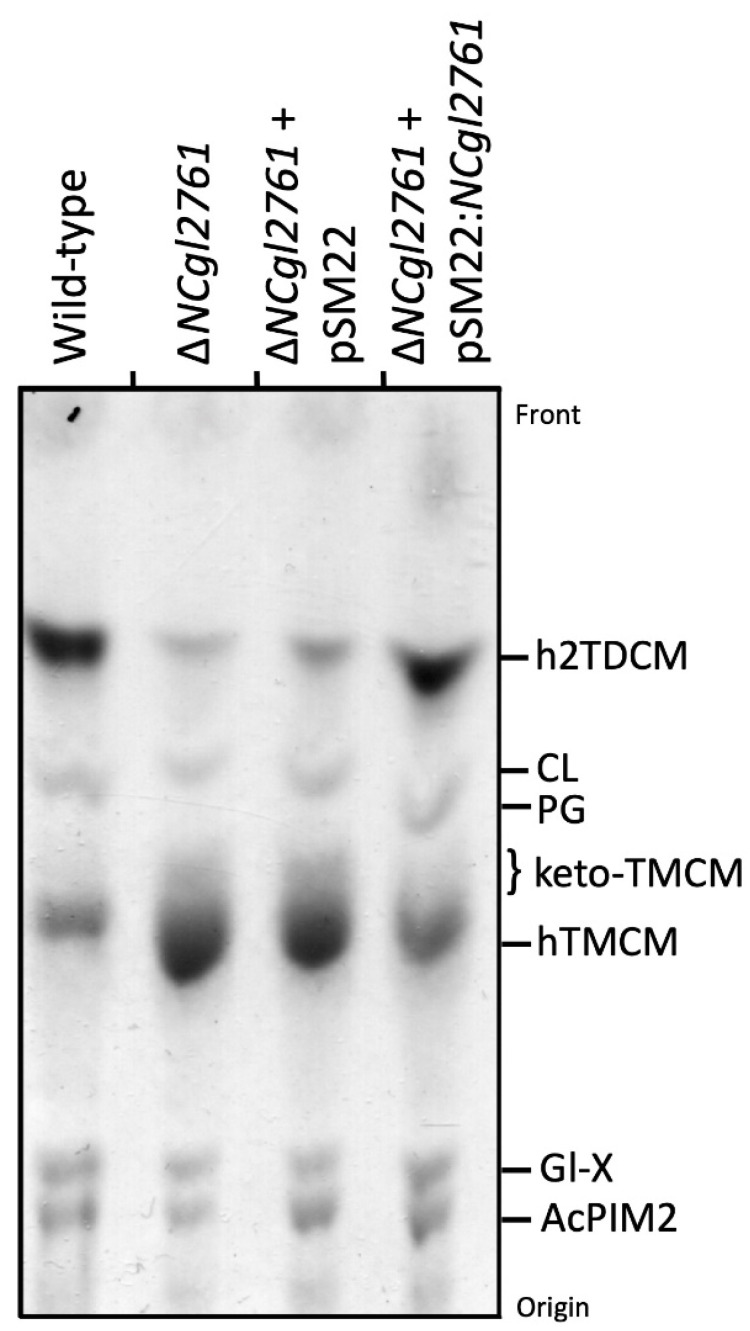
Cell wall free glycolipid analysis of a *C. glutamicum* Δ*NCgl2761* mutant. Lipids were extracted from WT *C. glutamicum*, Δ*NCgl2761*, Δ*NCgl2761* containing empty pSM22 and Δ*NCgl2761* containing pSM22:*NCgl2761*, then analysed by HPTLC. Glycolipids were visualized by orcinol-sulfuric acid staining. The identities of glycolipids indicated were based on previously published reports. CL, cardiolipin; PG, phosphatidylglycerol; Gl-X, Man.GlcA-DAG; AcPIM2, Man_2_-acyl-PI.

We next investigated whether *NCgl2761* is required for the synthesis of other apolar lipids such as the phosphatidyl-*myo*-inositol mannosides (PIM) or hTMCM/h2TDCM. Total glycolipids were extracted and analysed by HPTLC. Relative to WT bacteria, the Δ*NCgl2761* mutant line exhibited a marked increase in steady-state levels of hTMCM and a concomitant decrease in the synthesis of h2TDCM (Figure 4), although the occurrence of different h2TDCM molecular species was not affected (see Table 1). Levels of both TMCM and TDCM species were restored to WT levels following complementation with pSM22:*NCgl2761*, but not with empty pSM22. These analyses indicate that loss of *NCgl2761* is associated with a reduced rate of conversion of hTMCM to h2TDCM.

**Figure 5:**
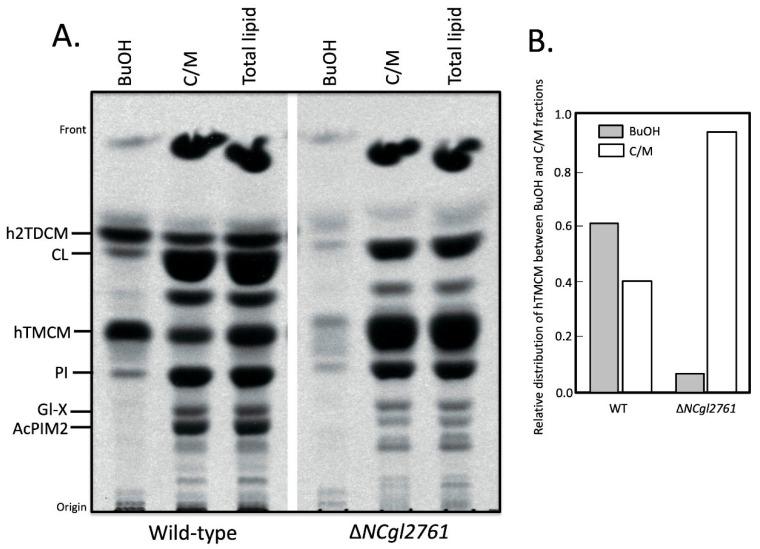
A *C. glutamicum* Δ*NCgl2761* mutant has a defect in TMCM surface transport. Wild-type and the Δ*NCgl2761* mutant were metabolically labelled with [^14^C]-acetate and sequentially extracted in 1-butanol (BuOH) and chloroform/methanol (C/M; 2:1 *v*/*v*). Parallel cultures were directly extracted in chloroform/methanol/water (Total lipid; 1:3:1 *v*/*v*). The three fractions from each bacterial line were analysed by HPTLC (**A**) and labelled species detected by fluorography and quantitated (**B**).

The accumulation of hTMCM in the Δ*NCgl2761* mutant could be explained by a defect in hTMCM transport across the IM for conversion to h2TDCM. We therefore investigated the subcellular distribution of hTMCM/h2TDCM in WT and the *NCgl2761* mutant using differential solvent extraction, as previously described [17]. Extraction of bacteria with 1-butanol results in the selective removal of OM lipids such as TDCM, but not cell (inner) membrane lipids such as PI. For WT cells, approximately 75% of the hTMCM labelled with [^14^C]-acetate was extracted by the 1-butanol, indicating that a major fraction of the steady- state pool of TMCM is located in the periplasmic space/OM (Figure 5). In contrast, <5% of the hTMCM was extracted by 1-butanol for the Δ*NCgl2761* mutant. Despite the reduced concentration of hTMCM in the OM, the occurrence of identified molecular species was not affected (see Table 1). These results support the hypothesis that loss of *NCgl2761* causes a defect in the transport of newly synthesized hTMCM in the IM to the periplasm/OM. 

### 3.6. A ΔNCgl2761 Mutant Phenocopies ΔtmaT and ΔmtrP Mutants

**Figure 6:**
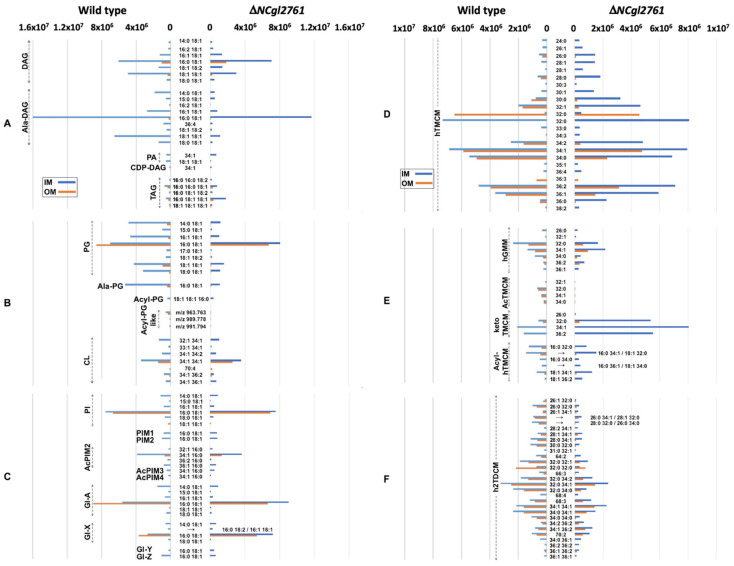
Comparison of the relative abundances of IM and OM lipids in *C. glutamicum* WT and a Δ*NCgl2761* mutant. The relative abundance of 144 species, identified by LC-MS/MS profiling, in the IM and OM fractions. Lipid abundances (based on ion intensities) represent the mean value of four replicates. The relative abundance of DAG and TAG based lipid classes (**A**), PG and CL-based lipid classes (**B**), PI/PIM and Gl glycolipid classes (**C**), hTMCM species (**D**), other trehalose and glucose corynomycolates (**E**) and h2TDCM species (**F**) are shown.

This hTMCM/h2TDCM phenotype is similar to that originally described for Δ*tmaT* (*NCgl2759*) [17,27] and Δ*mtrP* (*NCgl2764*) [18] mutants. We previously showed that loss of TmaT and MtrP led to global changes in the lipidomes of mutant lines [18,27]. To investigate whether similar changes also occurred in the Δ*NCgl2761* mutant line, we undertook a detailed analysis of the IM and OM lipid extracts of the WT and mutant strains by LC-ESI-QTOF-MS in positive ionization mode (Figure 6). Lipid species identified by MS/MS from WT and the Δ*NCgl2761* mutant are summarized in Table 1. After removal of isotopologues, dimer species, isomers, and isobars, 141 and 87 lipid species were identified with high confidence in the IM and OM of the Δ*NCgl2761* mutant, respectively. 

Significant changes were observed in the mutant line across several lipid groups, but particularly in lipids of the corynomycolate pathway (Figure 6; Table 1). Compared to WT bacteria, the IM fraction of the Δ*NCgl2761* mutant contained elevated levels of hTMCM and trehalose monoketocorynomycolates (keto-TMCM), the precursors of hTMCM. The IM to OM ratio for hTMCM in the WT was 1.24 but 12.92 for the *NCgl2761* mutant, while for keto-TMCM these ratios were 2.23 and 16.07, respectively (average values are shown and were calculated from all detected lipid species per sub-class; data not shown), confirming enrichment of both sub-classes in the IM. Overall, these findings suggest a reduced rate of membrane transport in the mutant, leading to a build-up of intermediates preceding the transport step. Short chain hTMCM species (<30 carbons) were significantly reduced in the OM but longer chain species were only mildly reduced, indicating less of an impact on the transport of species with >30 carbon chain lengths. A reduction in most h2TDCM species was observed, particularly in the OM fraction and for species with shorter carbon chains. Collectively, these data were consistent with our initial comparison of lipid extracts by HPTLC (Figure 4). 

A key finding of the lipidome analyses was that no AcTMCMs of any chain length were detected in the Δ*NCgl2761* mutant. These species were also absent in the *tmaT* mutant, as expected due to lack of a functional TmaT acyltransferase [17], but also in the *mtrP* mutant. In each mutant, the absence of AcTMCM correlated with a reduced rate of corynomycolate transport across the membrane, suggesting AcTMCM as a preferred transport substrate relative to hTMCM. Compared with WT bacteria, the IM fraction of the Δ*NCgl2761* mutant also lacked detectable Acyl-PG-like species and had decreased amounts of PG, Acyl-PG and Ala-DAG while the OM fraction was deficient in Acyl-PG, had decreased levels of PG and increased levels of hGMM. These changes were also observed in the *tmaT* (*NCgl2759*) and *mtrP* (*NCgl2764*) mutant lipidomes (Table 1), reflecting a common phenotype across all three lines. In contrast, PIM species and cardiolipins were generally unchanged in the mutant lines, indicating that some cell wall biosynthesis pathways remained unaffected by the absence of *NCgl2761*.

## 4. Discussion

**Figure 7:**
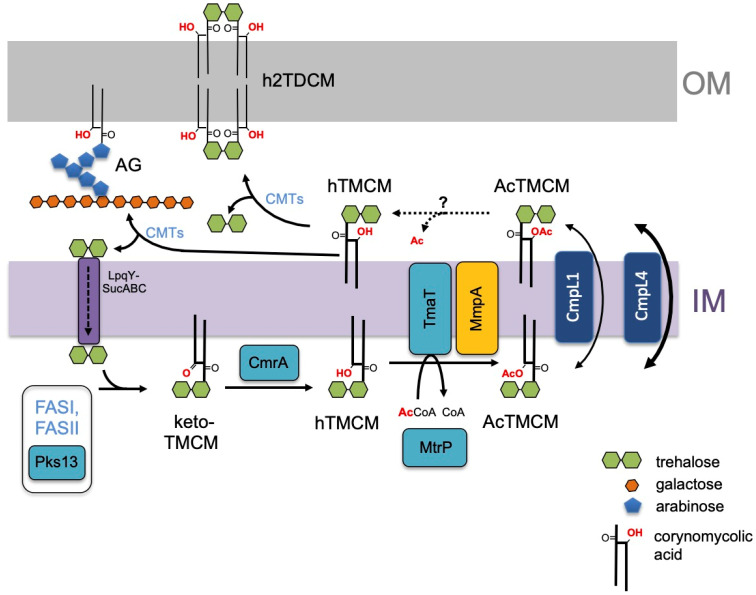
Proposed role of *C. glutamicum* MmpA in trehalose corynomycolate synthesis and transport. Corynomycolic acids, synthesized by the FAS-I and FAS-II pathways and Pks13, are linked to trehalose to form keto-TMCM and the keto group is subsequently reduced by CmrA reductase to form hTMCM. The hydroxylated corynomycolic acid in hTMCM is acetylated by the membrane acetyltransferase, TmaT, and AcTMCM transported across the IM by CmpL1 or CmpL4. AcTMCM is deacetylated by an unknown enzyme in the periplasmic space (dashed arrows) and hTMCM used as a corynomycolic acid donor by corynomycolyltransferases (CMTs) to synthesize h2TDCM or mycolated AG. Released trehalose is recycled by the LpqY-SucABC importer complex [32]. Additional acylation variants exist of hTMCM, AcTMCM and hTDCM [27]. Results of the current study place NCgl2761 (MmpA, orange) at the same step of the pathway as NCgl2759 (TmaT) [17] and NCgl2764 (MtrP) [18].

In this study, we show that *C. glutamicum* NCgl2761, designated here as MmpA, is required for the synthesis of AcTMCM and transport of TMCM species across the IM. *NCgl2761* is located in the middle of a gene locus that is largely conserved across all species of corynebacteria and mycobacteria. Two other genes in this locus have previously been shown to be involved in AcTMCM biosynthesis. *NCgl2759* encodes the membrane bound acetyltransferase, TmaT, that catalyses the addition of the acetyl group to the hydroxy-mycolic acid backbone of hTMCM. The second gene, *NCgl2764*, encodes a putative methyltransferase, MtrP, which lacks transmembrane domains or lipid modification motifs. Loss of *NCgl2764* also leads to a defect in AcTMCM synthesis, although the precise role of MtrP in the synthesis of this glycolipid remains undefined. In contrast to both *NCgl2759* and *NCgl2764*, *NCgl2761* is predicted to encode a protein which lacks recognizable catalytic domains or homology to known proteins. Strikingly, deletion of *NCgl2761* resulted in an essentially indistinguishable growth and biochemical phenotype as null mutants lacking *NCgl2759* or *NCgl2764*. In particular, all three mutants have primary defects in AcTMCM biosynthesis and transport of this glycolipid to the periplasmic space, with concomitant secondary effects on the synthesis of h2TDCM and other membrane lipids. Overall, this study strongly indicates that the proteins encoded by these three genes are functionally dependent on each other and/or act sequentially in the synthesis of AcTMCM (Figure 7). 

Orthologs of *C. glutamicum NCgl2761* are found within similar or identical gene loci in other Corynebacterium and Mycobacterium species, including *M. tuberculosis* (*Rv0226c*), *M. leprae* (*ML2582*), *M. bovis* (*Mb0231c*), *M. marinum* (*MMAR_0475*) and *M. smegmatis* (*MSMEG_0315*). The *M. tuberculosis* ortholog, Rv0226c, shares 26% amino acid sequence identity to NCgl2761 and has a similar domain structure. Rv0226c is reported to be essential for growth of *M. tuberculosis* H37Rv based on high density mutagenesis and sequencing studies [29,30]. Interestingly, while neither NCgl2761 nor Rv0226c share homology to protein outside the corynebacteria/mycobacteria, both NCgl2761/Rv0226c share partial homology to the N-terminal 668 residues of mycobacterial arabinofuranosyltransferase D (AftD) (data not shown). AftD is a 150 kDa, membrane-anchored transferase which adds the final α-linked arabinofuranose units to AG and LAM [33], although its precise role remains unclear. The cryo-EM structure of the *Mycobacterium abscessus* AftD has been solved to 2.9 Å resolution, revealing three carbohydrate-binding modules anchored in the membrane by sixteen transmembrane domains [34]. The portion of AftD that aligns with Rv0226c is devoted to membrane anchoring, as it comprises eleven of the sixteen transmembrane domains of the protein, with the C-terminal half of the protein containing the catalytic domains followed by the remaining five transmembrane helices. Rv0226c is also predicted to comprise eleven transmembrane domains, and this conserved topology along with the sequence similarity may indicate an evolutionary relationship between the two proteins, as well as a possible role for Rv0226c and its orthologs as membrane anchors for a protein complex. Interestingly, *Rv0226c* and the *M. tuberculosis* ortholog of *aftD*, *Rv0234c*, are separated by just nine genes.

Global lipidomic analysis of the *NCgl2759*, *NCgl2761* and *NCgl2764* null mutant lines support the notion that the primary function of these proteins is the regulation of AcTMCM synthesis and transport of TMCM across the IM. In particular, individual loss of each gene leads to the accumulation of keto-TMCM, the precursor of hTMCM [11], consistent with the back-up of hTMCM precursors, as well as the decrease in TDCM species that require the periplasmic transport of (Ac)TMCM for synthesis. However, these analyses also reveal broad changes in the composition of non-trehalose lipids in mutant lines. In particular, defects in AcTMCM were associated with supressed synthesis of phosphatidylglycerol (PG) and alanylated diacylglycerols (Ala-DAG), as well as the absence of a class of acyl-PG-like lipids. These changes could reflect compensatory changes in response to massive increase in TMCM in the inner leaflet of the cell membrane, and/or loss of TDCM in the outer membrane, as well as changes in the activities of other lipid biosynthetic enzymes in the cell membrane. 

It is notable that not all genes in the *NCgl2759* locus are involved in trehalose-lipid biosynthesis and trafficking. In particular loss of *NCgl2760*, the second gene in the locus, leads to defects in elongation of the macrolipoglycans, LM and LAM, while TMCM levels are unchanged [26]. Conversely, loss of *NCgl2759*/*NCgl2761*/*NCgl2764* (*tmaT*/*mmpA*/*mtrP*) has no effect on the maturation of LM and LAM. Together, these findings provide additional support for the conclusion that the three proteins are tightly linked functionally. Interestingly, *NCgl2761* and *NCgl2760* appear to be co-transcriptionally regulated, suggesting that the organization of these genes within a single genetic locus may provide a mechanism for regulating these two distinct pathways of cell wall biogenesis [26]. A degree of crosstalk between the pathways is not unexpected as (coryno)mycolates and LM/LAM are abundant, core components of mycobacterial/corynebacterial cell walls and their rates of synthesis must be coordinated to ensure the correct balance of both components is maintained during active cell growth and replication. 

Our findings are incorporated into a revised pathway for corynomycolate membrane transport in Figure 7. Based on the growth and lipid phenotypes of the Δ*tmaT* [17], Δ*mtrP* [18] and Δ*mmpA* mutant lines, we propose that the proteins encoded by *mtrP* and *mmpA* are required for activity of the TmaT acetyltransferase and/or act sequentially in regulating the synthesis of AcTMCM and transport of this intermediate across the membrane. Following transport and deacetylation by a yet to be identified enzyme, corynomycolates are processed by corynomycolyltransferases (CMTs) that combine from two hTMCM molecules to form h2TDCM or transfer the corynomycolates to acceptors on AG. A transport cycle is proposed based on the trehalose carrier, which is recycled back into the cytoplasm after releasing hTMCM. Further studies are required to define the relationships between these proteins and to identify additional proteins participating in the regulation of TMM/TMCM membrane transport in Corynebacterineae.

## Figures and Tables

**Table 1:** Detected lipid (sub)classes and lipid species of the IM and OM fraction of the *C. glutamicum* WT, Δ*tmaT* (Δ*NCgl2759*), Δ*mtrP* (Δ*NCgl2764*) and Δ*mmpA* (Δ*NCgl2761*) mutants via LC-MS/MS ESI TOF in positive ionization mode (CE30).

	Lipid (Sub)Class	WT		Δ*NCgl2759*		Δ*NCgl2764*		Δ*NCgl2761*	
		IM	OM	IM	OM	IM	OM	IM	OM
**1**	hTMCM	18	20	26	15	23	18	27	14
**2**	AcTMCM	3	8	0	0	0	0	0	0
**3**	keto-TMCM	3	0	10	3	8	5	6	4
**4**	Acyl-hTMCM	7	5	12	5	9	7	11	4
**5**	Acyl-AcTMCM	1	0	0	0	0	0	0	0
**6**	h2TDCM	28	28	30	25	27	29	28	26
**7**	Ac1-hTDCM	3	3	3	1	0	0	0	0
**8**	hGMM	5	11	8	7	8	8	7	5
**9**	DAG	7	4	8	4	7	5	7	4
**10**	Ala-DAG	11	5	9	2	7	1	5	1
**11**	CDP-DAG	1	0	1	0	1	1	0	1
**12**	TAG	10	10	11	11	11	10	13	8
**13**	PG	11	5	5	2	6	5	6	2
**14**	Acyl-PG	3	0	1	0	1	0	1	0
**15**	Ala-PG	2	1	1	0	1	1	0	1
**16**	PA	2	0	0	0	1	1	1	1
**17**	CL	16	3	14	4	10	5	9	5
**18**	PI	4	3	3	2	4	5	4	4
**19**	PIM1	1	1	1	0	1	1	1	0
**20**	PIM2	1	1	1	1	1	1	1	1
**21**	AcPIM2	4	1	5	1	4	1	4	1
**22**	AcPIM3	1	0	1	0	1	1	1	1
**23**	AcPIM4	1	0	1	0	1	0	0	0
**24**	Gl-A	6	2	5	1	3	2	4	1
**25**	Gl-X	6	1	4	1	3	2	3	1
**26**	Gl-Y	1	0	1	1	0	1	1	1
**27**	Gl-Z	1	0	1	0	1	1	1	1
**28**	Acyl-PG-like	4	3	0	0	0	0	0	0
	**SUM:**	**161**	**115**	**162**	**86**	**139**	**111**	**141**	**87**

## Data Availability

Mass spectrometry data available on request.

## Supplementary Materials

The following are available online at https://www.mdpi.com/article/10.3390/biom11121760/s1, Figure S1: Amino acid sequence alignment of the *C. glutamicum* ATCC 13032 protein NCgl2761 and putative orthologs in *M. tuberculosis* H37Rv (Rv0226c), *M. smegmatis* mc^2^ 155 (MSMEG_0315) and *M. leprae* (ML2582); Figure S2: Lipoglycan analysis of a *NCgl2761* mutant.
