# Supplementary material for: MmpA, a Conserved Membrane Protein Required for Efficient Surface Transport of Trehalose Lipids in Corynebacterineae
Source: Biomolecules. 2021 Nov 24;11(12):1760. doi: 10.3390/biom11121760 (PMC8698533; doi:10.3390/biom11121760)
Supplement: Supplementary file 1 [file biomolecules-11-01760-s001.zip › biomolecules-1460438-supplementary.pptx]

## Slide 1
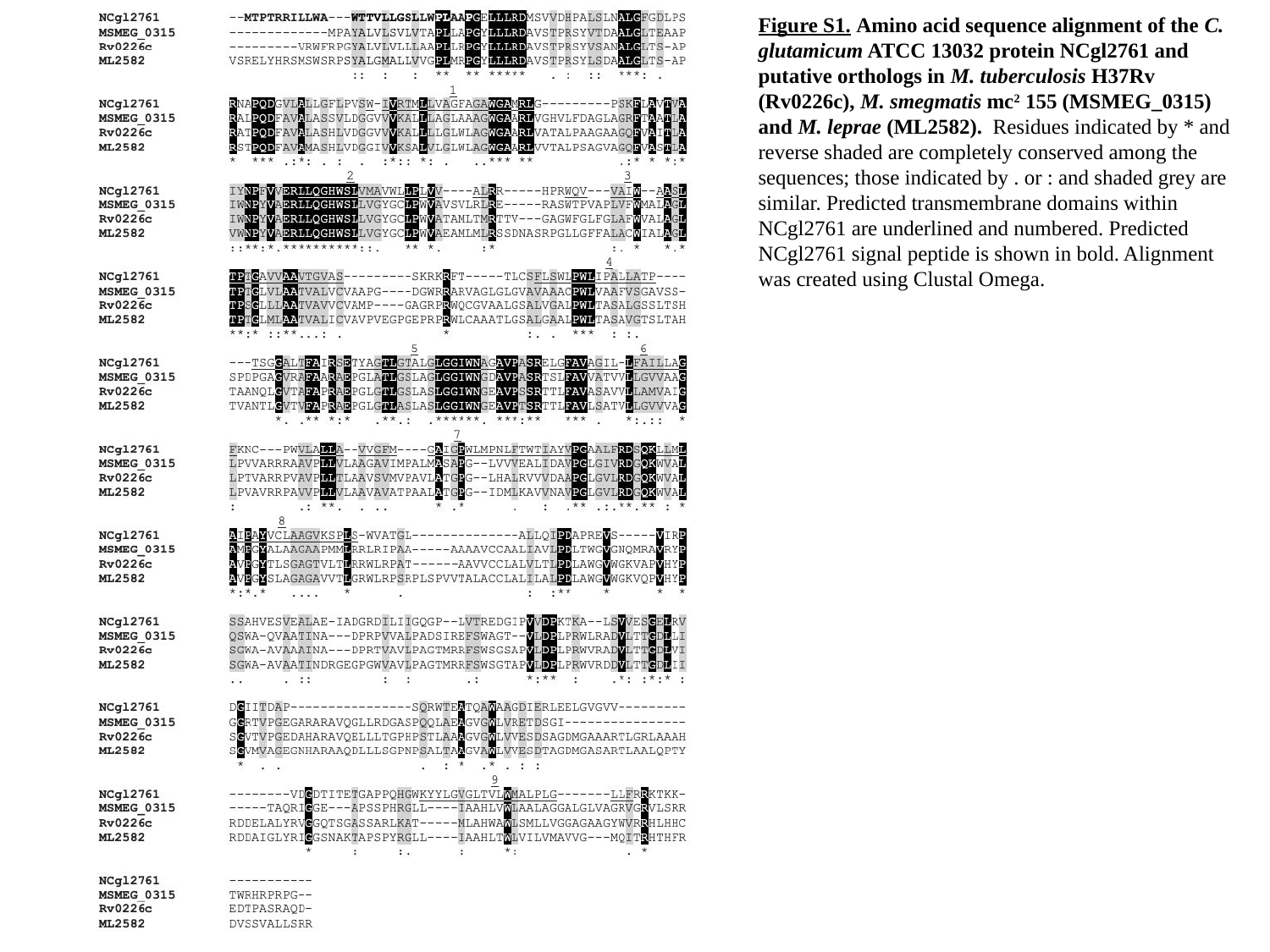

Figure S1. Amino acid sequence alignment of the C. glutamicum ATCC 13032 protein NCgl2761 and putative orthologs in M. tuberculosis H37Rv (Rv0226c), M. smegmatis mc2 155 (MSMEG_0315) and M. leprae (ML2582). Residues indicated by * and reverse shaded are completely conserved among the sequences; those indicated by . or : and shaded grey are similar. Predicted transmembrane domains within NCgl2761 are underlined and numbered. Predicted NCgl2761 signal peptide is shown in bold. Alignment was created using Clustal Omega.

## Slide 2
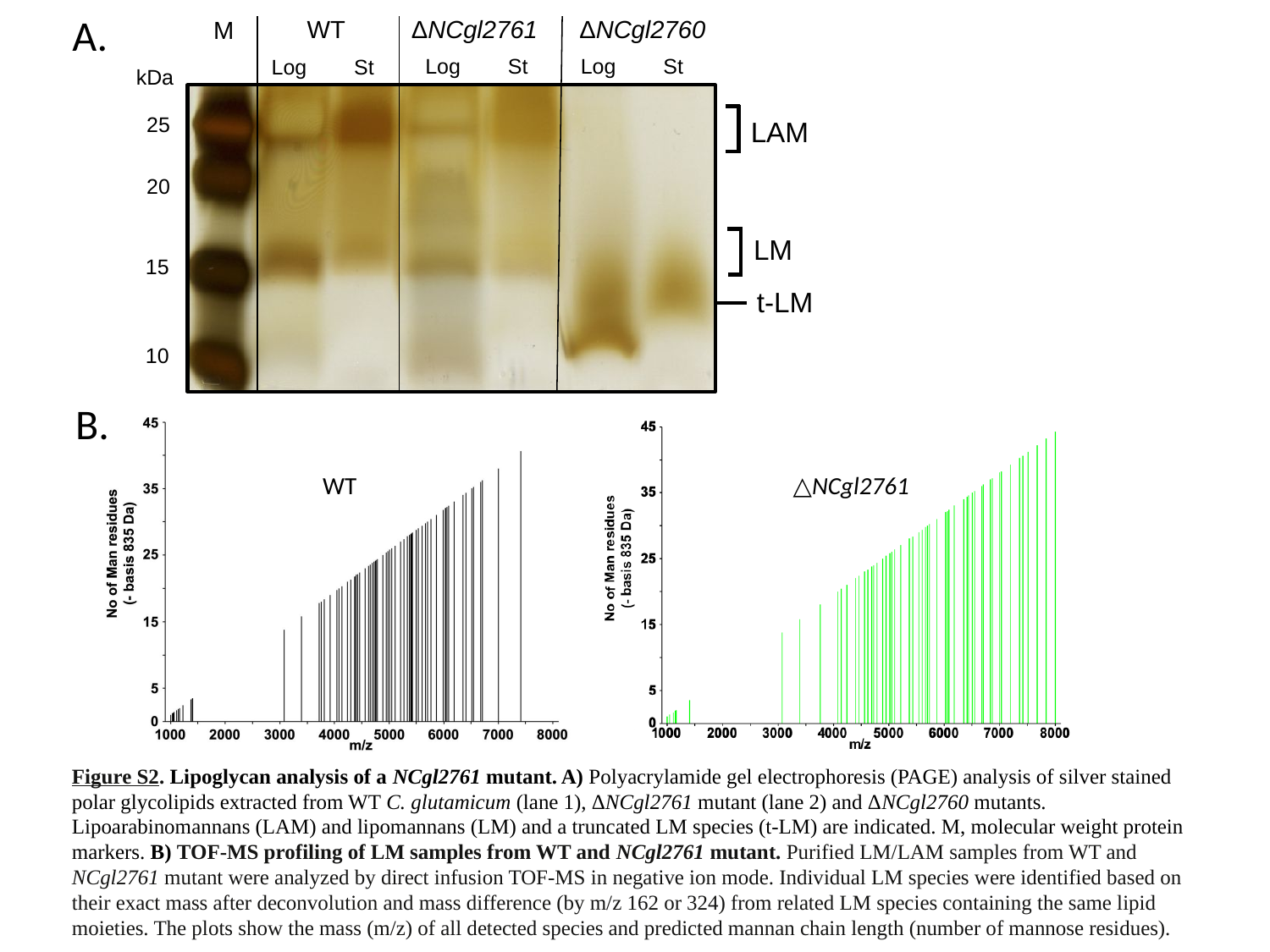

A.
WT
ΔNCgl2761
ΔNCgl2760
M
Log St
Log St
Log St
kDa
25
LAM
20
LM
15
t-LM
10
B.
WT
△NCgl2761
Figure S2. Lipoglycan analysis of a NCgl2761 mutant. A) Polyacrylamide gel electrophoresis (PAGE) analysis of silver stained polar glycolipids extracted from WT C. glutamicum (lane 1), ΔNCgl2761 mutant (lane 2) and ΔNCgl2760 mutants. Lipoarabinomannans (LAM) and lipomannans (LM) and a truncated LM species (t-LM) are indicated. M, molecular weight protein markers. B) TOF-MS profiling of LM samples from WT and NCgl2761 mutant. Purified LM/LAM samples from WT and NCgl2761 mutant were analyzed by direct infusion TOF-MS in negative ion mode. Individual LM species were identified based on their exact mass after deconvolution and mass difference (by m/z 162 or 324) from related LM species containing the same lipid moieties. The plots show the mass (m/z) of all detected species and predicted mannan chain length (number of mannose residues).
